# Supplementary material for: Transient dynamics of the phase transition in VO2 revealed by mega-electron-volt ultrafast electron diffraction
Source: Nat Commun. 2023 Mar 7;14:1265. doi: 10.1038/s41467-023-37000-2 (PMC9992676; doi:10.1038/s41467-023-37000-2)
Supplement: Supplementary file 1 — Supplementary Information [file 41467_2023_37000_MOESM1_ESM.pdf]

Supplementary Information

Transient dynamics of the phase transition in VO_2 revealed by mega-electron-volt ultrafast electron diffraction

Chenhang Xu[1], Cheng Jin[2,3], Zijing Chen[2,3], Qi Lu[1], Yun Cheng [2,3], Bo Zhang[1], Fengfeng Qi [2,3], Jiajun Chen[1], Xunqing Yin[1], Guohua Wang[1], Dao Xiang[2,3,4,5,*] and Dong Qian,[1,4, 6,*]

[1]Key Laboratory of Artificial Structures and Quantum Control (Ministry of Education), Shenyang National Laboratory for Materials Science, School of Physics and Astronomy, Shanghai Jiao Tong University, Shanghai 200240, China

[2]Key Laboratory for Laser Plasmas (Ministry of Education), School of Physics and Astronomy, Shanghai Jiao Tong University, Shanghai 200240, China

[3]Collaborative Innovation Center of IFSA, Shanghai Jiao Tong University, Shanghai 200240, China

[4]Tsung-Dao Lee Institute, Shanghai Jiao Tong University, Shanghai 200240, China

[5]Zhangjiang Institute for Advanced Study, Shanghai Jiao Tong University, Shanghai 200240, China

[6]Collaborative Innovation Center of Advanced Microstructures, Nanjing 210093, China

[*] email: dxiang@sjtu.edu.cn; dqian@sjtu.edu.cn

Supplementary Note 1. Epitaxial relationship and morphology of VO_2 films

Supplementary Note 2. Simulation on the TEM diffraction pattern of VO_2 film

Supplementary Note 3. Thermally-induced structural phase transition of VO_2 films

Supplementary Note 4. Lattice dynamics after photoexcitation below and above threshold

Supplementary Note 5. Peak width of 200 spot in UED

Supplementary Note 6. Thresholds at different temperatures

Supplementary Note 1. Epitaxial relationship and morphology of VO_2 films

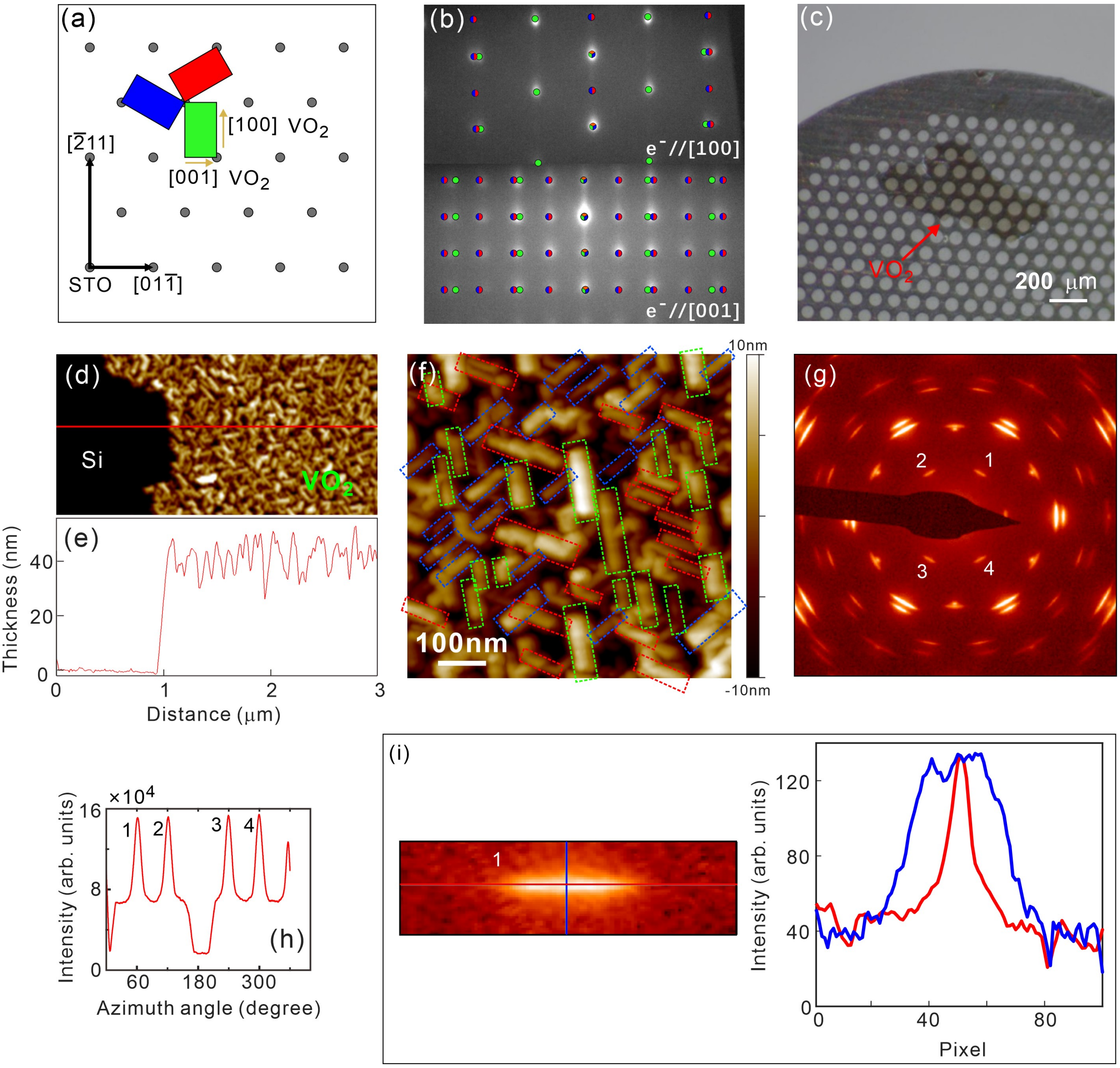

Supplementary Figure 1. (a) Epitaxial relationship of VO_2 (010) on STO (111). Green/red/blue rectangles represent three orientations (or called domains). (b) RHEED patterns during the growth overlaid with the simulated patterns. Upper: incident electrons are along the [100] direction of the green VO_2 domain in (a), Bottom: incident electrons are along the [001] direction of the green VO_2 domain. Green/red/blue colors represent diffraction spots from three domains in (a). (c) Optical image of the freestanding 40 nm VO_2/2 nm STO on the TEM grid. (d) AFM topography of a VO_2/2-nm STO film transferred on a Si wafer to check the thickness. (e) AFM line profiles along the red line in (d). (f) High resolution AFM topography of a VO_2/2-nm STO film. Grains with an in-plane anisotropic shape were observed. There are three orientations indicated by red, green and blue rectangles. (g) TEM pattern with electron beam incident along [010] direction. The diffraction region is 4 μm in diameter.

(h) Intensity distribution of diffraction spots, labelled as 1 – 4 in (g), along the azimuthal direction. (i) Line profiles of spot No. 1 along two directions.

The epitaxial growth of VO_2 on STO (111) surface was studied in the previous report (ref. 47 in the main text). Because VO_2 (010) plane has a 2-fold symmetry and STO (111) surface has a 6-fold symmetry, there are three possible in-plane orientations with an interval of 120° (called 120° domains, for convenience), as shown in Supplementary Fig. 1(a). For instance, in the green domain, the epitaxial relationships are: $[001](010)_{VO2}//[01\bar{1}](111)_{STO}$ and $[100](010)_{VO2}//[\bar{2}11](111)_{STO}$. The best lattice match is between VO_2 [101] and STO $[\bar{1}10]$. The mismatch is about -2.5%. Supplementary Fig. 1(b) shows the RHEED pattern in the R phase. Simulated RHEED patterns considering three 120° domains were overlaid on the experimental results. The experimental results are in excellent agreement with simulations.

Supplementary Fig. 1(c) shows the optical image of the freestanding films on the TEM grid. The size of this sample is about $0.3\ mm \times 0.7\ mm.$ The electron beam size in UED is about 0.15 mm in diameter. Supplementary Fig. 1(d) shows the AFM topography of a VO_2 film transferred onto a Si substrate. The roughness of this film is about 6 nm. According to the AFM line profile in Supplementary Fig. 1(e), the film thickness is about 40 nm. High resolution AFM topography is shown in Supplementary Fig. 1(f). There are lots of crystal grains, but only three orientations were observed, indicated by red, green and blue rectangles, which is consistent with Supplementary Figs. 1(a) and (b). All grains have an in-plane anisotropic shape, which is caused by the different lattice mismatch between VO_2 and STO along $[001]_{VO2}$ and $[100]_{VO2}$ directions. Grains prefer to grow larger along the direction with less lattice mismatch. For most grains, the ratio of the short side to the long side is between 1:3 to 1:4, and the grain size (long side) is between $50 \sim 150$ nm.

Supplementary Fig. 1(g) shows the diffraction pattern measured by 200 keV TEM with a diffraction area of 4 μm in diameter. By comparing with the spot of STO, we obtained the average in-plane lattice constant is about 5.4 ± 0.1 Å^{-1}. In this rea, the distribution of domains should be uniform in average. Supplementary Fig. 1(h) show the intensity distribution of diffraction spots, labeled # 1, 2, 3, 4 in Supplementary Fig. 1(g), along the azimuthal direction. According to our simulation (Supplementary Fig. 1(d)), each spot in # 1 - 4 consist of contributions from two 120° domains. Clearly, the intensities of spots 1 - 4 are almost the same, indicating the equal contribution of the domains within the diffraction area. Supplementary Fig. 1(i) shows the line profiles of spot # 1 along two orthogonal directions. The peak width (FWHM) along the elongation direction is about six times larger than that along the perpendicular direction. Roughly speaking, the elongated shape (ratio between two directions is ~ 1:6) of the spot #1 is compatible with the anisotropic shape of grains (ratio is ~ 1:3 to 1:4), when considering that spot #1 consists of two diffraction spots (Supplementary Fig. 1(d)).

Supplementary Note 2. Simulation on the TEM diffraction pattern of VO_2 film

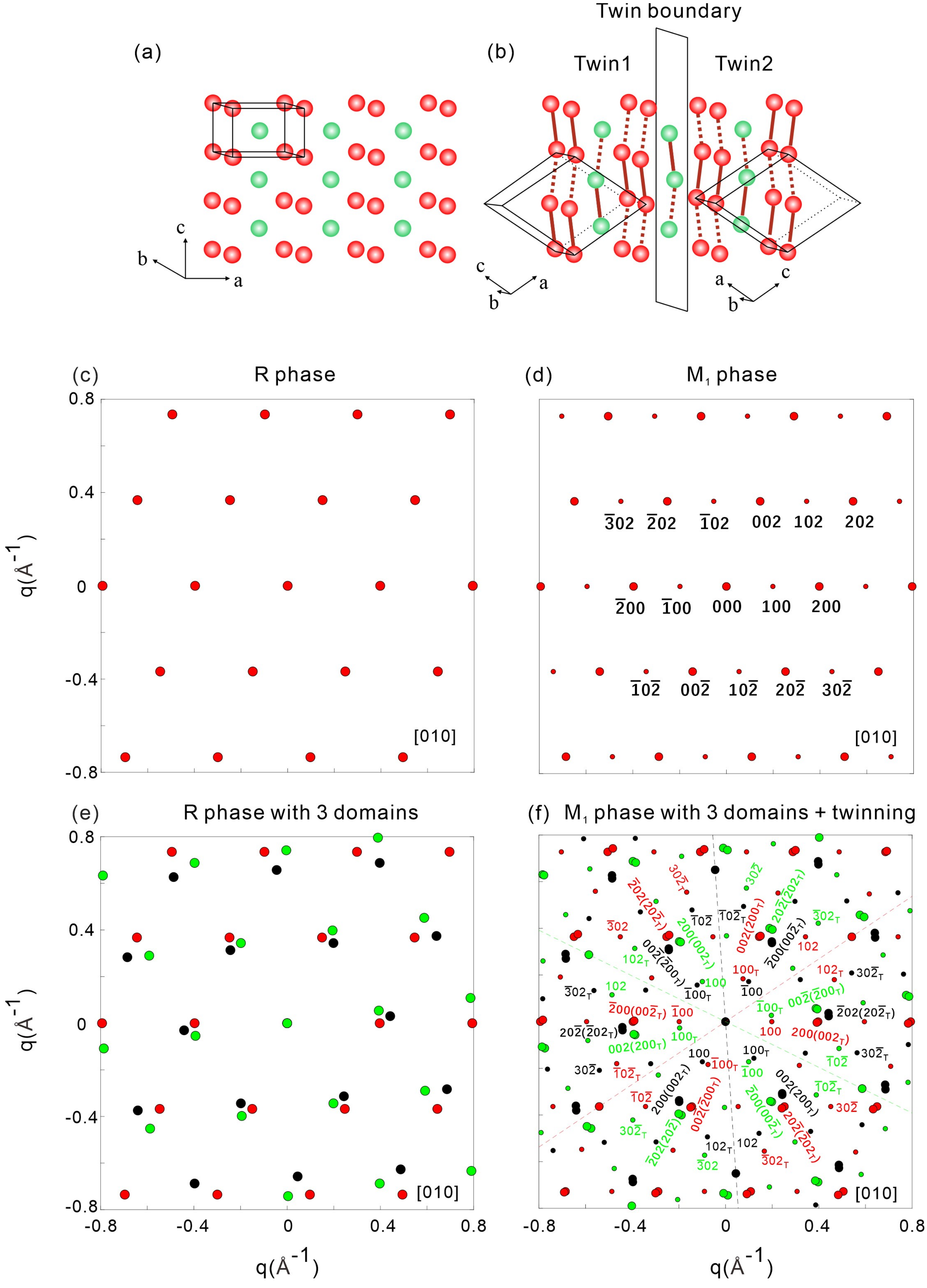

Supplementary Figure 2. Sketches of the crystal structure of VO_2 in (a) R-phase and (b) M_1-phase. Only V atoms are presented. Solid red line represents the dimerized V−V bonds. Simulated TEM diffraction pattern in (c) R-phase, (d) M_1-phase (Twin2), (e) R-phase with three domains, and (f) M_1-phase with three domains and twinning. Different colors represent the contributions from different 120° domains. Subscript "T" represents spots from a twin domain. The incident electrons are along VO_2 [010] direction. For convenience, we use smaller dots to present the superstructure spots.

Crystal structures of VO_2 in the R-phase and the M_1-phase are shown in Supplementary Figs. 2(a) and (b). Only V atoms are presented for simplicity. Solid red lines represent the dimerized bonds. Sketched in Supplementary Fig. 2(b), twin domains can form in the M_1-phase. Simulated TEM diffraction patterns for a perfect single crystalline VO_2 in the R-phase and the M_1-phase are presented in Supplementary Figs. 2(c) and (d), respectively. Miller Indexes are based on M_1-phase representation. M_1-phase has the superstructure spots (small spots in Supplementary Fig. 2(d)) due to the V−V dimers. The simulated pattern for the R-phase with three 120° domains is shown in Supplementary Fig. 2(e). Red/green/black colors correspond to different domain. When VO_2 is in the M_1 phase, each 120° domain will have twin domains. As a result, there will be $3 \times 2 = 6$ domains. Supplementary Fig. 2(f) shows the simulated pattern in the M_1 phase with 120° domains and twin domains.

Supplementary Note 3. Thermally-induced structural phase transition of VO_2 films

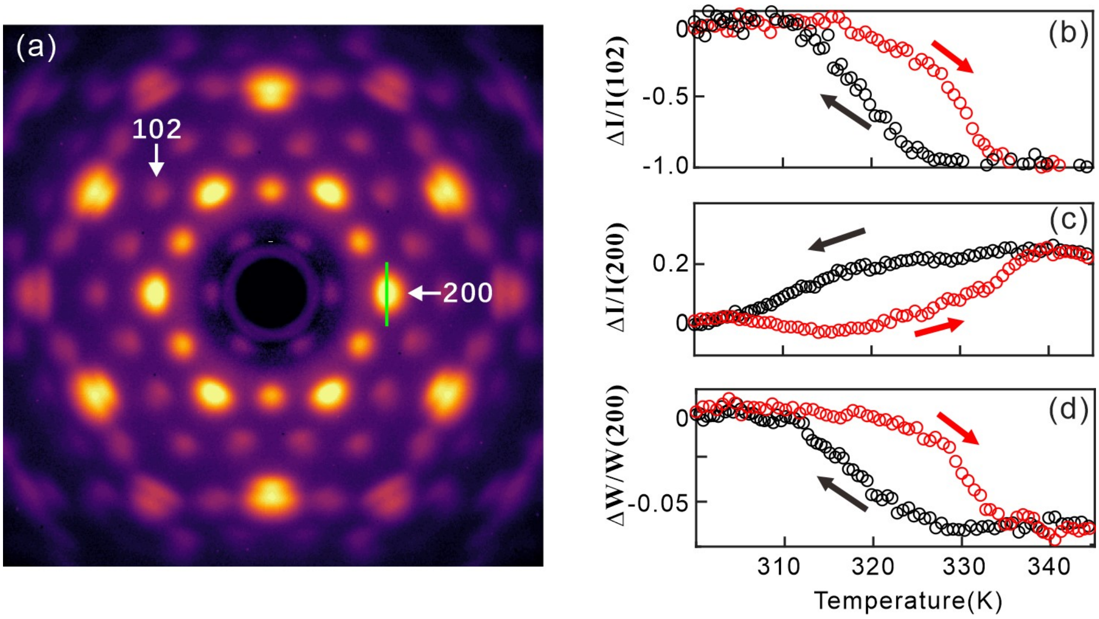

Supplementary Figure 3. (a) Static electron diffraction pattern measured with 3-MeV UED at 300K. (b) Temperature dependence of intensity change of 102 spot in thermal equilibrium. (c) Temperature dependence of the intensity change of 200 spot. (d) Temperature dependence of the peak width change along the green line direction of 200 spot. All the measurements are in thermal equilibrium.

Supplementary Fig. 3(a) is the static diffraction pattern measured by the 3-MeV UED system. We determined the structure phase transition temperature by tracking the evolution of the superstructure spot 102 as a function of temperature. Upon increasing the temperature, the intensity of 102 spot begins to decrease at about 315 K and drop to zero at about 335 K. Consistent with the resistance measurements for VO_2, a temperature hysteresis also exists for the diffraction intensity. We define the temperature where intensity starts to decrease as the structural phase transition temperature. For our films, $T_c \approx 315$ K. It should be noted that T_c of bulk VO_2 single crystal is 65℃ [1], nearly 20 K higher than our sample. This difference may result from the oxygen defect in our sample.

Supplementary Note 4. Lattice dynamics after photoexcitation below and above threshold

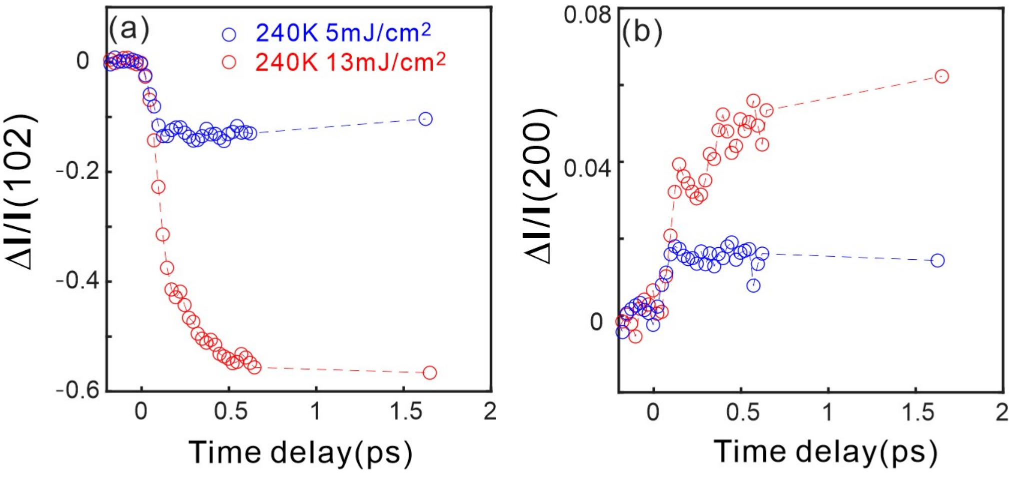

Supplementary Figure 4. Time evolution of intensity change of (a) 102 and (b) 200 peaks with the pump laser fluences below and above the threshold.

Shown in Supplementary Fig. 4, the intensity change of 102 and 200 peaks starts to recover at a time delay longer than 1.5 ps under a low laser fluence (5 mJ/cm^2). Under a high laser fluence (13 mJ/cm^2), intensity of two peaks does not recover, which is consistent with the photo induced structural phase transition.

Supplementary Note 5. Peak width of 200 spot in UED

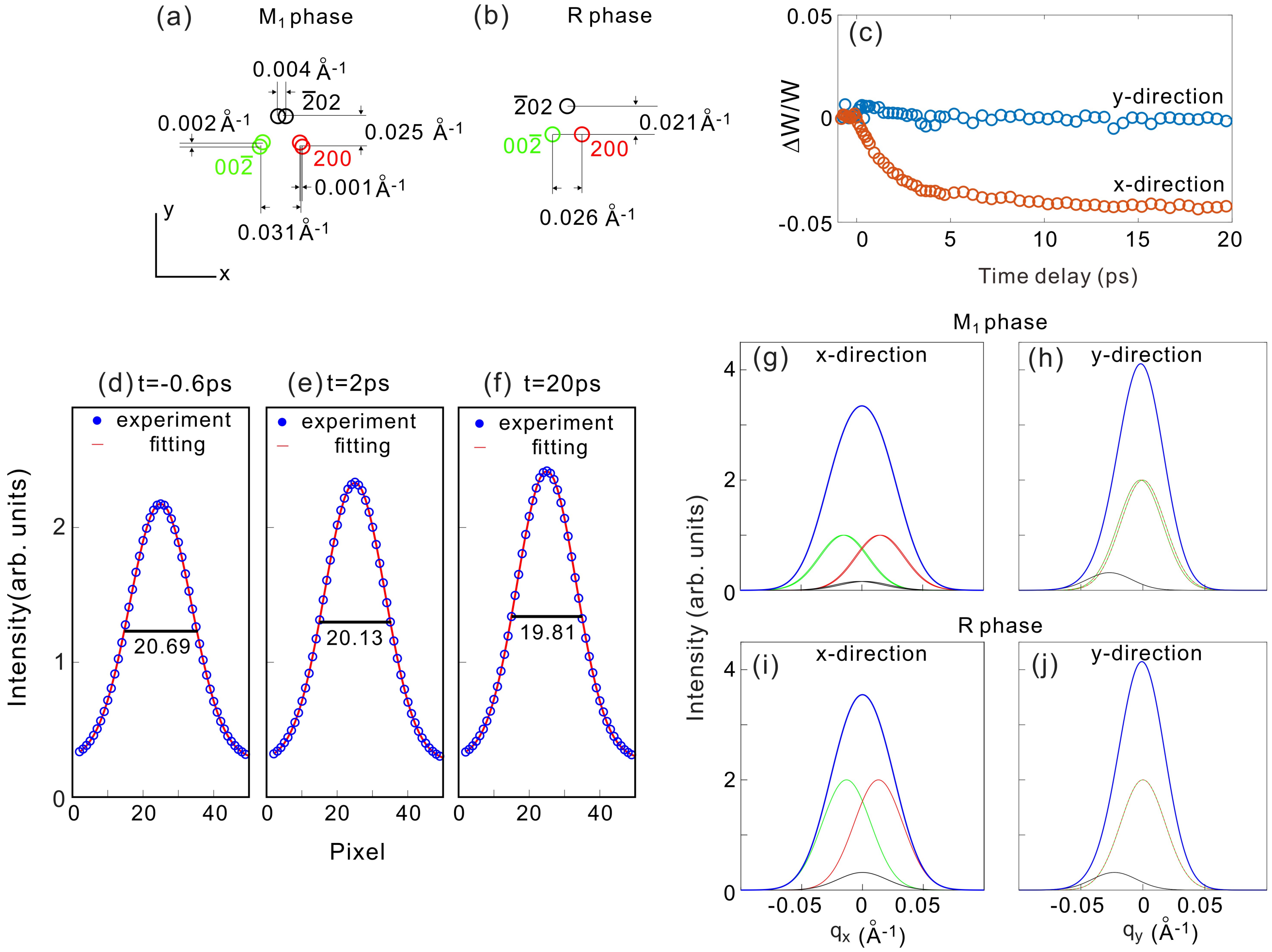

Supplementary Figure 5. (a) Distribution of the six spots inside the big "200" spot in the M_1 phase. (b) Distribution of the three spots inside the big "200" spot in the R phase. (c) The evolution of peak width of the big "200" spots after photoexcitation along two directions. Experimental data and Gaussian fitting along x-direction at (d) t = -0.6 ps, (e) t = 2 ps, and (f) t = 20 ps. Sketches of the line profiles of the six spots in the M_1 phase along (g) x-direction, and (h) y-direction. Blue line is the superposition of the six spots. Sketches of the line profiles of the three spots in the R phase along (i) x-direction, and (j) y-direction. Blue line is the superposition of the three spots.

As shown in Figs. 1(d) and 2(a) in the main text, the big "200" spots in the M_1 phase consist of six spots. They are $00\bar{2}$, 200, $\bar{2}02$ and their twins. We plot these six spots in Supplementary Fig. 5(a). The distances between different spots are calculated. Shown in Supplementary Fig. 5(a), the distance between $00\bar{2}$ and 200 is ~ 0.031 Å^{-1} along x-direction, which is the same as the green line in Fig. 2(a) in the main text. The splitting of $\bar{2}02$ due to the twin domains is ~ 0.004 Å^{-1} along x-direction. The splitting of $00\bar{2}$ (200) due to the twin domains has both x and y components. It is ~ 0.002 Å^{-1} along x-direction and ~ 0.001 Å^{-1} along y-direction. The distance between $\bar{2}02$ and $00\bar{2}$ (200) is

$\sim 0.025\,\text{Å}^{-1}$ along y-direction. In the R phase, twin domains disappear. As a result, the six spots become three spots, as shown in Supplementary Fig. 5(b). Meanwhile, $00\bar{2}$, 200, and $\bar{2}02$ will also move closer. The distance between $00\bar{2}$ and 200 along x-direction becomes $\sim 0.026\,\text{Å}^{-1}$. The distance between $\bar{2}02$ and $00\bar{2}$ (200) becomes $\sim 0.021\text{Å}^{-1}$ along y-direction.

Experimentally, benefited from the excellent signal-to-noise ratio, we obtained the evolution of the peak width of the big "200" spot as a function of time delays along x and y directions, shown in Supplementary Fig. 5(c). Supplementary Figs. 5(d)-(f) show the experimental data and Gaussian fitting along x-direction at t = - 0.6 ps, t = 2 ps, and t = 20 ps, respectively. At t = 2 ps, the peak width reduces by about 0.5 pixel. The measurement uncertainty depends on the diffraction signal, stability of incident electrons, exposure time and number of measurements. In our case, we captured a diffraction pattern with an exposure time of two seconds at each time delay and repeated 45 times. Then we averaged the diffraction patterns at each time delay to get the final diffraction pattern. The width measured at a fixed time delay averaged over forty-five measurements yields an uncertainty of about 0.13 pixel that corresponds to 0.0005A^{-1}.

Interestingly, the evolution of peak width is very anisotropic. Peak width along y-direction is nearly constant. This anisotropic behavior can be understood by Supplementary Figs. 5(g)-(j). Supplementary Figs. 5(g) and (h) present the line profiles of the six spots in the M_1 phase (Supplementary Fig. 5(a)) along x and y-direction, respectively. The peak height is set according to the structure factor of each spot. It should be noted that the intensity of $\bar{2}02$ spot is about 1/6 of $00\bar{2}$/200 spots according to the structure factor. To compare with our data, we need to add up the intensities of the six spots to get the final line profile (blue line). For simplicity, the peak width is set to the same for all the six spots and let the superposed line profile has the similar peak width as the experimental result. Supplementary Figs. 5(i) and (j) present the line profiles of the three spots in the R phase (Supplementary Fig. 5(b)) along x and y-direction, respectively. Blue lines are the superposition.

For x-direction, it is clear that both the twin domains splitting of $00\bar{2}$/002/$\bar{2}02$ and the separation between $00\bar{2}$ and 002 will contribute to peak width since the splitting and the separation both are along x-direction. In the R phase, twin domains disappear, $00\bar{2}$ and 002 move closer. Therefore, we can observe peak width narrowing along x-direction. By fitting the blue lines in Supplementary Figs. 5(g) and (i), we can obtain a relative change of peak width of about -6%. It must be noted that the simulation is more qualitative than quantitative. Nevertheless, it is comparable with the experimental value, i.e. ~ - 5%, in Supplementary Fig. 5(c).

We can do another rough estimation. The distance between 200 and $00\bar{2}$ is ~ 0.031 Å^{-1} in the M_1 phase. It reduces by ~ 0.005 Å^{-1} in the R phase. If we assume the total peak width in the M_1 phase is ~ 0.031 Å^{-1} and take 0.005 Å^{-1} as the change of width, then we get the largest relative change of peak

width of about -16%. In real experiments, the total peak width of the diffraction spots becomes much larger because of the crystal quality and beam size. The relative change of the peak width must be much less than -16%. In fact, the experimental peak width is about 0.07 $Å^{-1}$ in our samples, corresponding to a relative change of about -7%. In our experiments, the extracted relative change of peak width after transition from the M_1 phase to R phase is about -5%.

For y-direction, it is very different. The separation between $00\bar{2}$ and 200, as well as the twin domain splitting of $\bar{2}02$ peak does not contribute to the change of peak width along y-direction, because such separation and splitting are along x-direction. The y-component of the twin domain splitting of $00\bar{2}$ and 200 spots can contribute to the change of peak width, however it is very small. On the other hand, $\bar{2}02$ spot will move closer to $00\bar{2}$/200 spots along y-direction during the transition from the M_1 to R phase, which could reduce the peak width in principle. However, as seen in Supplementary Figs. 5(h) and (j), $\bar{2}02$ peak is much weaker than $00\bar{2}$/200 peak and can only contribute to the left tail of the overall line profile. Therefore, when the line profile is fitted by a Gaussian peak, the obtained peak width is nearly unaffected by the movement of $\bar{2}02$ peak. As a result, the change of the peak width along the y-direction should be much weaker than that along the x-direction. By fitting the blue lines in Supplementary Figs. 5(g) and (i), we can obtain a relative change of peak width of about - 0.5 %, which is consistent with the observed result.

Supplementary Note 6. Thresholds at different temperatures

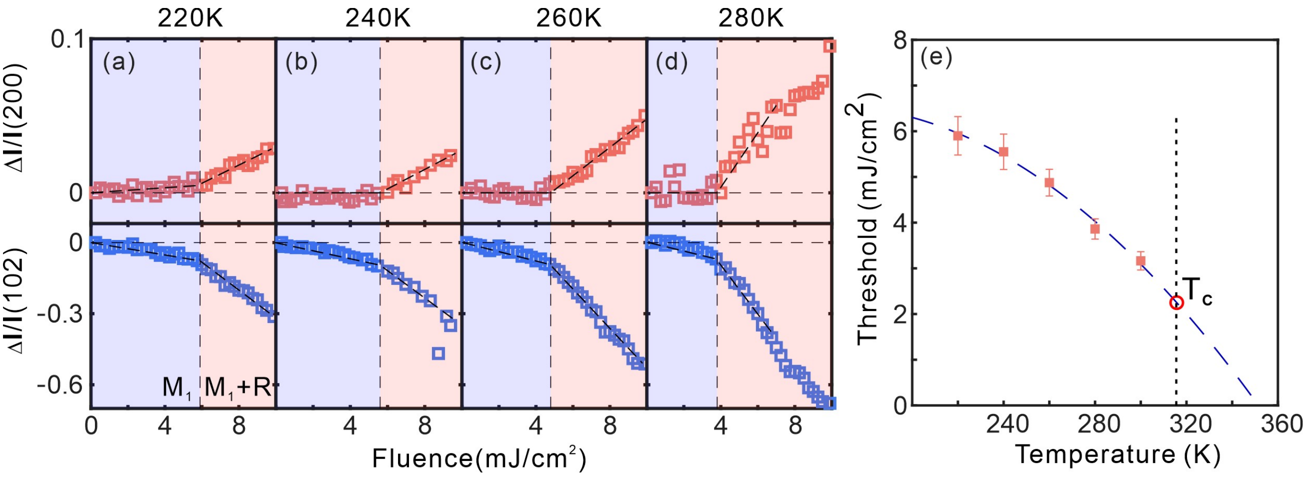

Supplementary Figure 6. Fluence dependence of intensity change for 102 peaks and 200 peaks at 10 ps after optical pump (a) 220K, (b) 240K, (c) 260K and (d) 280K. (e) Experimental measured laser fluence thresholds as a function of the sample temperature. Blue dashed line is the fitting curve. Error bars in (e) is generated by calculating the standard deviation of different measurements at the same condition.

Supplementary Fig. 6 presents the fluence thresholds extracted from the intensity change for 200 and 102 peaks at different temperatures. The kink at low temperatures is more abrupt than that at 300 K, which may be related to some local structure transition as the temperature is close to Tc. The laser thresholds are determined as ~ 5. 9 mJ/cm^2 at 220 K, ~ 5.6 mJ/cm^2 at 240 K, ~ 4.8 mJ/cm^2 at 260 K and ~ 3.8 mJ/cm^2 at 280 K. Assuming the threshold in VO_2 is thermally dominated, the total thermal energy required to drive the phase transition can be described by $E_{th} = \int C_V(T)dT + H_L$, where C_V is heat capacity and H_L is latent heat. According to previous report [1], the heat capacity of VO_2 increases approximately linearly from 220 K to 300 K, which means that the thermal energy needed to drive the phase transition should decrease quadratically. We found that the experimental data can be fitted by a quadratic curve, yielding a latent heat of ~ 2.2 mJ/cm^2 at T_C = 315 K, in good agreement with that obtained by THz spectroscopy [2]. The fact that the thresholds obtained from the same sample at different temperatures fit quite well with a quadratic curve suggests that the magnitude of the threshold is likely thermally driven. Heat can be quickly transferred from the photoexcited electrons to the lattice and SPT occurs once the thermal barrier is crossed.

Supplementay References

[1] C. N. Berglund and H. J. Guggenheim, Electronic Properties of VO2 near the Semiconductor-Metal Transition, Phys. Rev. 185, 1022 (1969).

[2] A. Pashkin, C. Kübler, H. Ehrke, R. Lopez, A. Halabica, J. Haglund, R. F., R. Huber, and A. Leitenstorfer, Ultrafast insulator-metal phase transition in VO_2 studied by multiterahertz spectroscopy, Phys. Rev. B 83, 195120 (2011).
